# Comparative Efficacy and Cost-Effectiveness of Denosumab Versus Zoledronic Acid in Cancer Patients with Bone Metastases

**DOI:** 10.3390/jcm14186469

**Published:** 2025-09-14

**Authors:** Vali Aliyev, Murad Guliyev, Murat Günaltılı, Mehmet Cem Fidan, Emir Çerme, Hamza Abbasov, Zeliha Birsin, Selin Cebeci, Nebi Serkan Demirci, Özkan Alan

**Affiliations:** Department of Internal Medicine, Division of Medical Oncology, Cerrahpaşa Faculty of Medicine, Istanbul University-Cerrahpaşa, Istanbul 34098, Türkiye

**Keywords:** bone metastases, skeletal-related events (SREs), denosumab, zoledronic acid, cost-effectiveness, cancer

## Abstract

**Background:** This retrospective study compared the effectiveness and preliminary cost evaluation of denosumab and zoledronic acid (ZA) in patients with bone metastases from breast, prostate, and lung cancer. **Methods:** Patients treated with ZA or denosumab between January 2016 and August 2023 were analyzed. Outcomes included the incidence of skeletal-related events (SREs), time to first SRE, and cost per prevented SRE. An incremental cost-effectiveness analysis (ICER framework) was also performed, using prevention of SREs as the effectiveness outcome. **Results:** A total of 192 patients in the denosumab group and 239 in the ZA group were included. Denosumab significantly reduced the incidence of SREs compared with ZA (34.8% vs. 51.8%, *p* < 0.001). The median time to first SRE was longer with denosumab (34.5 vs. 29.1 months), but the difference was not statistically significant (*p* = 0.593). Stratified analyses showed significant benefit in breast (29.5% vs. 49.2%, *p* = 0.002) and prostate cancer (43.9% vs. 66.0%, *p* = 0.035), but not in lung cancer (39.1% vs. 45.9%, *p* = 0.484). Denosumab was more costly, with an additional USD 4686 per prevented SRE. **Conclusions:** Denosumab was more effective than ZA in reducing SREs, particularly in breast and prostate cancer patients, but it was associated with higher costs. These findings should be interpreted as preliminary due to the retrospective design and the absence of QALY-based outcomes.

## 1. Introduction

Bone metastases and related complications represent a significant cause of morbidity in advanced solid and hematologic cancers. They are the most common site of metastasis, especially in breast, prostate and lung cancers [1,2]. Skeletal-related events (SREs) associated with bone metastases may include pathologic fracture, spinal cord compression, radiation, or surgery to bone. These events dramatically impair patients’ quality of life and increase healthcare costs due to the necessity of additional interventions such as surgery, radiation therapy and hospitalization [3]. Bone-modifying agents (BMAs) play a key role in reducing both the frequency and severity of SREs in individuals with bone metastases [4].

Bisphosphonates and osteoclast inhibitors, such as denosumab, are widely used in the treatment of patients with bone metastases. Zoledronic Acid (ZA), a potent bisphosphonate, has been demonstrated to be more effective than other bisphosphonates in delaying initial SREs [2]. However, denosumab, a fully human monoclonal antibody which inhibits the RANK ligand (RANKL), has emerged as a promising alternative. Evidence from randomized clinical trials suggests that denosumab delays skeletal-related events more effectively than zoledronic acid in breast and prostate cancer, while providing comparable outcomes in lung cancer [5,6,7,8,9,10,11,12]. An additional advantage of denosumab is its suitability for patients with impaired renal function, whereas zoledronic acid requires dose modifications in this context [13]. Despite these clinical benefits, denosumab remains substantially more costly than ZA, and this cost difference continues to raise concerns about its cost-effectiveness, particularly in low- and middle-income settings [14].

Both ZA and denosumab demonstrate a favourable tolerance profile; however, they are both linked to significant adverse effects. The prevalent adverse effects include medication-related osteonecrosis of the jaw (MRONJ) and hypocalcemia. Denosumab has been reported to have a lower incidence of MRONJ compared to ZA, which may be considered a clinical advantage. Nonetheless, denosumab has been associated with a reduced occurrence of renal toxicity in comparison to ZA, making it a more appropriate option for patients with pre-existing renal conditions [15]. However, a rebound phenomenon characterized by increased bone turnover and a higher risk of SREs after denosumab discontinuation has also been described and should be considered in long-term treatment planning.

In this study, we aimed to retrospectively analyze the efficacy of denosumab and ZA in patients with metastatic lung, breast and prostate cancers. We also assessed the cost-effectiveness of these treatments in preventing SREs.

## 2. Materials and Methods

### 2.1. Study Design

Within the study population, patients older than 18 years of age with metastatic lung, breast, and prostate cancer (denosumab is not covered by insurance in our country for other types of cancer) who received ZA or denosumab treatment for bone metastases between January 2016 and August 2023 were retrospectively analyzed. The choice between denosumab and ZA was based on reimbursement policies, treating physician’s clinical judgment, and patient-specific factors such as renal function or treatment accessibility. Inclusion criteria consisted of patients with a histopathologically confirmed diagnosis of breast, lung, or prostate cancer, with at least one bone lesion demonstrated on imaging techniques such as computed tomography (CT; Siemens Healthineers, Erlangen, Germany), positron emission tomography (PET; GE Healthcare, Chicago, IL, USA), or magnetic resonance imaging (MRI; Philips Healthcare, Best, The Netherlands), who had received a minimum of 2 months of therapy and had a follow-up period of at least 6 months. We excluded patients who had previously received other BMA agents, underwent less than 6 months of follow-up, or had inadequate clinical documentation.

### 2.2. Patient Population

We collected clinicopathological data from patient medical charts and institutional electronic databases. Baseline variables included age, sex, Eastern Cooperative Oncology Group (ECOG) performance status, presentation as de novo or recurrent metastatic disease, history of SREs before BMA initiation, metastatic sites, visceral versus non-visceral involvement, and treatment-related adverse events. Twenty-seven patients were excluded due to incomplete follow-up (*n* = 14), prior treatment with other BMAs (*n =* 8), or insufficient records (*n =* 5). All eligible patients had an ECOG performance status of 2 or below, preserved organ function, baseline creatinine clearance ≥ 30 mL/min, and had received BMA therapy for at least two months.

For bone metastases, zoledronic acid (Zometa^®^, Novartis Pharma AG, Basel, Switzerland) was administered at a dose of 4 mg intravenously every four weeks, either at a standard dose or adjusted according to creatinine clearance following the established clinical practice of our center. Denosumab (Xgeva^®^, Amgen Inc., Thousand Oaks, CA, USA) was administered at a dose of 120 mg subcutaneously every four weeks at a standard dosage. In breast and lung cancer patients, bone-modifying agents were routinely initiated shortly after the diagnosis of bone metastasis, independent of the systemic therapy protocol. In prostate cancer, treatment with bone-modifying agents was introduced during the castration-resistant phase, in accordance with ESMO Clinical Practice Guidelines on Prostate Cancer, which define metastatic castration-resistant prostate cancer (mCRPC) [16].

### 2.3. Efficacy and Safety Measures

SREs were defined as pathologic fracture, spinal cord compression, or the need for radiation therapy or surgical intervention to bone. Time to first SRE after BMA was defined as the time from the start of BMA treatment to the occurrence of the first SRE. Censoring was performed at the last follow-up for patients who remained free of SREs. Hypocalcemia, drug-associated osteonecrosis of the mandible and renal failure, which were more common as specific side effects, were examined. Treatment-related adverse events (TRAEs) were classified according to the National Cancer Institute Common Terminology Criteria for Adverse Events (CTCAE), version 5.0 [17], which defines SREs as pathological fractures, spinal cord compression, or need for radiation/surgery to bone due to metastases. Therapeutic oral or intravenous calcium was administered to patients who developed hypocalcemia during BMA treatment.

In our country, the cost of a single administration of denosumab is $105, whereas the cost of zoledronic acid is $22 per dose. Cost calculations were based on these prices. The cost analysis conducted in the study was performed using the Number Needed to Treat (NNT) method.

### 2.4. Ethical Considerations

The research protocol was conducted in line with the ethical principles of the Declaration of Helsinki and was approved by the institutional ethics committee (5 March 2025; reference number: E-74555795-050.04-1260324). Owing to its retrospective design, the need for informed consent was waived.

### 2.5. Statistical Analysis

Statistical analyses included descriptive methods, with continuous variables summarized as mean ± standard deviation, median, and range, while categorical variables were expressed as frequencies and percentages. Group comparisons were made using Fisher’s exact or Chi-square tests for categorical variables, and independent *t*-tests for continuous variables. Logistic regression analyses (both univariate and multivariate) were conducted to evaluate risk factors for SRE development. Time-to-SRE was assessed using multivariable Cox proportional hazards regression, adjusted for key baseline characteristics (tumor type, ECOG performance score, previous SRE, and visceral metastases). Odds ratios (ORs) and 95% confidence intervals (CIs) were calculated. Kaplan–Meier survival curves and log-rank tests were used to compare time to first SRE between treatment arms. To minimize bias related to follow-up differences, a sensitivity analysis truncated follow-up at 37 months, corresponding to the median observation period in the denosumab group. Two-tailed *p* values < 0.05 were defined as statistically significant.

All statistical analyses were performed using SPSS Statistics for Windows, version 27.0 (IBM Corp., Armonk, NY, USA).

## 3. Results

### 3.1. Characteristics of Patients

Our study included 431 patients and 262 (60.7%) female patients with a median age of 57 years (range: 24–88). There were 192 (44.5%) patients in the denosumab group and 239 (55.5%) patients in the ZA group. There were 233 (54.1%) patients with breast cancer, 107 (24.8%) with lung cancer (75 men, 70.1%), and 91 (21.1%) with prostate cancer. The median follow-up time was 47 months (range: 6.1–328.3 months). Baseline demographic, clinicopathologic and treatment initiation times were similar between the two groups (Table 1).

Prior to the start of denosumab and ZA treatment, 52.6% and 58.2% of patients had an SRE, respectively. In both groups, the most common SRE was palliative radiotherapy to the bone, with rates of 50.5% and 54.8%, respectively. The median follow-up time was 37 months (range: 6.1–250.1 months) for the denosumab group and 56 months (range: 6.1–328.3 months) for the ZA group. The median duration from the diagnosis of bone metastasis to the initiation of bone-targeted therapy was 56 days (range: 0–3844 days).

At follow-up, 67 patients (34.8%) receiving denosumab developed an SRE compared to 124 patients (51.8%) receiving ZA (*p* < 0.001). In patients treated with BMAs, the denosumab group demonstrated a numerically longer median time to first SRE than the ZA group; however, this difference was not statistically significant: 34.5 months (95% CI: 25.9–45.1) and 29.1 months (95% CI: 20.5–37.8), respectively (*p* = 0.593). In both groups, the most common SRE was palliative radiotherapy to the bone, with rates of 94.0% and 91.1%, respectively.

In a sensitivity analysis truncating follow-up at 37 months (the median follow-up of the denosumab arm), 34 (21.6%) denosumab-treated patients and 42 (20.1%) ZA-treated patients developed SREs. The Kaplan–Meier cumulative incidence at 37 months did not differ significantly between the two groups (log-rank χ^2^ = 0.133, *p* = 0.716), and Cox regression yielded a hazard ratio of 1.40 (ZA vs. denosumab, *p* = 0.149) (see Supplementary Table S3).

When stratified by tumor type, comparing denosumab and ZA groups, the first SRE development was in 31 (29.5%; 95% CI 21.6–38.8) versus 63 (49.2%; 95% CI 40.7–57.8) in breast cancer patients (*p* = 0.002), 18 (43.9%; 95% CI 29.9–59.0) versus 33 (66.0%; 95% CI 52.2–77.6) in prostate cancer patients (*p* = 0.035), and 18 (39.1%; 95% CI 26.4–53.5) versus 28 (45.9%; 95% CI 34.0–58.3) in lung cancer patients (*p* = 0.484).

**Figure 1:**
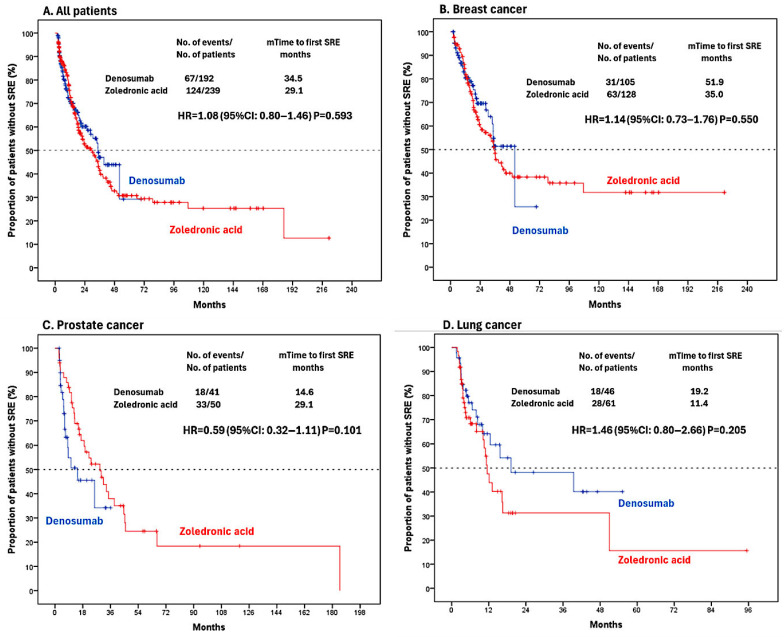
Kaplan–Meier estimates of time to the first post-BMA SRE by treatment group. (**A**) All patients. (**B**) Breast cancer subgroup. (**C**) Prostate cancer subgroup. (**D**) Lung cancer subgroup. (The dashed line represents the median time to first SRE).

In the denosumab and ZA groups, the median time to first SRE after BMA therapy was 51.9 versus 35 months for breast cancer (*p* = 0.550), 14.6 versus 29.1 months for prostate cancer (*p* = 0.101), and 19.2 versus 11.4 months for lung cancer (*p* = 0.205) (Figure 1).

**Figure 2:**
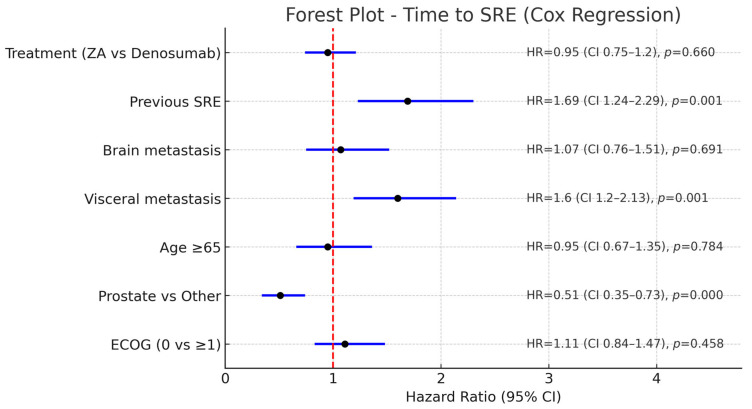
Forest plot of multivariate Cox regression analysis for time to SRE. (The vertical dashed red line represents the reference value (HR = 1.0)).

In the multivariate Cox regression analysis for time to SRE, previous SRE history (HR: 1.69, 95% CI: 1.24–2.30, *p* < 0.001) and visceral metastases (HR: 1.60, 95% CI: 1.16–2.21, *p* = 0.004) were associated with an increased risk of SRE, whereas prostate cancer as the primary tumor was associated with a lower risk compared with other primaries (HR: 0.51, 95% CI: 0.35–0.73, *p* < 0.001). Treatment type, age, brain metastases, and ECOG performance status were not significantly associated with time to SRE (Figure 2).

The effects of age, ECOG PS, treatment agent, primary cancer subtype, presence of SRE before treatment, and visceral and cranial metastases on the risk of developing SRE have been assessed using logistic regression analysis. Presence of SRE before treatment, cancer subtype, and treatment agent were found to be significantly associated with the risk of developing SRE. We found that the risk of developing SRE in patients treated with ZA was 2 times higher than in those treated with denosumab [OR: 2, 95% CI: 1.3–2.9, *p* = 0.001]. Tumor type emerged as a significant predictor; compared with prostate cancer, other primaries were associated with an almost two-fold higher risk of SRE (OR = 1.9, 95% CI: 1.22–3.19, *p* = 0.005). The logistic regression model of factors affecting the risk of developing SRE is shown in Table 2.

Overall, denosumab treatment was associated with shorter median treatment durations (11 vs. 17 months, *p* < 0.001) but significantly higher median costs ($1155 vs. $374, *p* < 0.001) compared to ZA. In breast cancer, the median treatment duration for denosumab was 17 months, while for ZA it was 22 months (*p* = 0.002). In terms of cost, denosumab was associated with a median cost of $1680, compared to $484 for ZA (*p* < 0.001). In prostate cancer, the median treatment duration for denosumab was 7 months, compared to 21 months for ZA (*p* < 0.01). The costs were $735 and $451, respectively (*p* < 0.01). In lung cancer, the median treatment duration for denosumab was 9 months, compared to 5 months for ZA (*p* = 0.084), but the costs were significantly higher for denosumab (median $945 vs. $110, *p* < 0.001) (Table 3).

### 3.2. Number Needed to Treat Analysis

**Figure 3:**
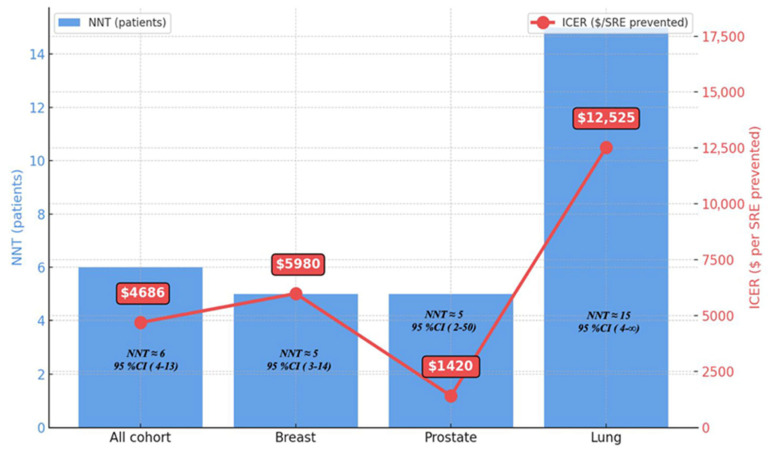
Incremental cost per prevented skeletal-related event (ICER Framework) in denosumab versus Zoledronic Acid. ($, USD).

In our cohort, it was estimated that approximately six additional patients would need to be treated with denosumab rather than ZA to prevent one patient from experiencing a first SRE. This corresponded to an additional cost of $4686 per prevented SRE. For breast cancer and prostate cancer, an additional five patients were required, with a cost of $5980 and $1420, respectively. In lung cancer, an additional fifteen patients were required, with a cost of $12,525 (Figure 3).

Due to adverse effects, treatment was discontinued in 4 (2%) of patients in the denosumab group and 14 (6%) in the ZA group. Adverse events included MRONJ in 3 patients (2%) in the denosumab group versus 5 patients (2%) in the ZA group, grade 3 hypocalcemia in 7 patients (4%) in the denosumab group versus 2 patients (1%) in the ZA group, and grade 3 renal toxicity observed in 3 patients (1%) in the ZA group, whereas no renal toxicity was reported in the denosumab group. MRONJ incidence was similar in both groups, hypocalcaemia was more common in the denosumab group (*p* = 0.001).

## 4. Discussion

Our results showed that denosumab was significantly more effective than ZA in preventing SREs, especially in breast and prostate cancer patients. On the other hand, no statistically meaningful difference was observed between the two treatments in lung cancer patients. These results represent unadjusted comparisons and therefore indicate associations rather than causality; the findings should be interpreted in the context of potential residual confounding despite adjustment in multivariable analyses. Denosumab showed better efficacy but higher costs compared to ZA; therefore, its cost-effectiveness appears context-dependent, being more favorable in breast and prostate cancer but limited in lung cancer due to non-significant clinical benefit and substantially higher cost per event prevented.

Stopeck et al. and Fizazi et al. reported that denosumab was superior to ZA in the delay of SREs in breast and prostate cancer patients, respectively [5,6]. Similarly, a previous meta-analysis of three phase III trials by Lipton et al. confirmed that compared with ZA, denosumab significantly reduced the risk of first SRE and also delayed the time to first SRE [18]. When stratified by cancer type, denosumab showed a significant reduction in SREs in breast and prostate cancer patients, whereas no significant benefit was observed in lung cancer patients. This lack of statistical difference is likely explained by the aggressive disease course and the shorter survival times in lung cancer, which limit the observation of long-term benefits from bone-modifying agents. Our results are in line with previous studies, including Scagliotti et al. [19], which also reported no significant advantage of denosumab over ZA in lung cancer patients. Our findings were consistent with the existing literature. However, the lung cancer subgroup in our cohort was relatively small and underpowered; therefore, these results should be interpreted with caution and considered exploratory.

Previous studies have demonstrated that denosumab is superior to ZA in delaying the time to first SRE across all primary cancer subgroups [20,21,22,23]. Consistent with these findings, our study showed that denosumab prolonged the time to first SRE in patients with lung and breast cancer compared to ZA; however, this superiority was not observed in patients with prostate cancer. The lack of observed benefit in the prostate cancer subgroup may be explained by the predominantly osteoblastic nature of bone metastases, limitations inherent to retrospective analyses, and differences in the underlying biological mechanisms across tumor types. Taken together, the lack of significant differences in some subgroups can be attributed both to disease biology—such as the aggressive course of lung cancer and the predominantly osteoblastic pattern of prostate cancer metastases—and to methodological limitations of our study, including its retrospective design and the reduced statistical power of subgroup analyses.

Studies have shown that denosumab is more effective in reducing SREs, but not cost-effective compared to ZA, as the higher costs of denosumab are not superior to the similar benefits observed with ZA [24,25,26,27,28,29]. A study by Body et al. (2015) found that denosumab reduced the risk of SREs by 17% compared to ZA, with a hazard ratio of 0.83 (95% CI: 0.76–0.90) [30]. Similarly, a cost-effectiveness analysis by Stopeck et al. (2012) concluded that denosumab was cost-effective in breast cancer patients but not in prostate cancer patients due to higher treatment cost and relatively less reduction in SREs in this population [31]. These findings highlight the importance of tailoring treatment decisions to individual patient characteristics, including cancer type, renal function, and economic considerations.

In our analysis, logistic regression identified prior SRE, non-prostate primaries, and zoledronic acid treatment as independent predictors of SRE. While logistic regression is suitable for binary outcomes, it does not account for event timing. Therefore, we also performed a Cox regression for time-to-SRE, which confirmed prior SRE and visceral metastases as adverse prognostic factors, and prostate cancer as protective. These consistent results support the robustness of our findings, although Cox regression remains the more appropriate approach for time-dependent outcomes.

In terms of cost-effectiveness, denosumab was associated with higher costs than ZA; however, since our analysis did not include QALY-adjusted ICER measures, these findings should be regarded as preliminary. Based on our data, denosumab showed better efficacy but higher costs compared with ZA. Its cost-effectiveness appeared more favorable in breast and prostate cancer, whereas in lung cancer the high number needed to treat and substantially increased cost per prevented SRE suggest limited economic value. These results are consistent with previous cost-effectiveness analyses, such as Stopeck et al. and Arellano et al., which highlighted the higher cost of denosumab despite its superior efficacy [14,31]. Our cost-effectiveness analysis considered both direct treatment costs and clinical benefit in terms of reduced SRE incidence. In addition, due to differences in follow-up duration between the groups, we performed a sensitivity analysis truncated at 37 months, which showed no significant difference in SRE incidence between denosumab and ZA.

Our cost-effectiveness analysis considered both direct treatment costs and clinical benefit in terms of reduced SRE incidence. In addition, we performed an incremental cost-effectiveness analysis (Figure 3), which demonstrated the incremental cost per prevented SRE. This analysis represents a simplified ICER framework, with SRE prevention serving as the effectiveness outcome. While this provides a more robust economic comparison between denosumab and ZA, we acknowledge that the absence of QALY-based outcomes limits the comprehensiveness of our evaluation. Therefore, future cost-effectiveness studies should incorporate QALY-adjusted ICER analyses to fully capture both economic and patient-centered outcomes.

Recent regulatory developments highlight the growing importance of denosumab biosimilars. In 2025, the FDA approved biosimilars from Fresenius Kabi for use in all Prolia^®^ and Xgeva^®^ indications. Other companies, including Sandoz, Celltrion, and Samsung Bioepis, have also submitted biosimilars for approval in the US and EU. These products are expected to improve access and reduce treatment costs significantly [32]. As biosimilars become integrated into clinical practice, treatment costs are expected to decrease substantially, which may improve the cost-effectiveness profile of denosumab. Future analyses should therefore re-evaluate denosumab in the context of biosimilar availability.

The incidence of osteonecrosis of the jaw was identical in both groups (2%), consistent with the 1–3% range reported in major trials [33,34]. As expected, grade 3 hypocalcemia was significantly more frequent with denosumab (4% vs. 1%, *p* = 0.001), mirroring the known mechanism of RANKL inhibition [34]. In some studies, renal toxicity was observed at a rate of 3–8% in patients treated with ZA [7], while in our study, grade 3 renal toxicity showed the expected pattern with 1% and was not seen in the denosumab group, which reinforced the renal safety advantage of denosumab [35]. Although this study did not assess post-treatment effects, previous research has reported a rebound phenomenon after denosumab discontinuation, characterized by increased bone turnover and vertebral fractures. Sequential bisphosphonate therapy has been proposed to reduce this risk [36].

The limitations of our study included its retrospective design, which may introduce selection bias and confounding factors. Additionally, the follow-up duration differed between treatment groups, which could have influenced the outcomes. Although propensity score matching was not performed, we attempted to minimize potential imbalances by conducting multivariate analyses and a sensitivity analysis truncated at 37 months, both of which consistently supported the robustness of our findings. Another limitation is that treatment allocation between ZA and denosumab was partly influenced by reimbursement policies and insurance coverage in our country, rather than being based solely on clinical randomization. In addition, the exact timing of pre-treatment SREs was not available in our dataset; therefore, only their presence or absence could be analyzed. Moreover, the absence of QALY-based data limited our ability to conduct a comprehensive cost-utility analysis. Although we performed an incremental cost-effectiveness analysis to provide a simplified ICER framework using SRE prevention as the outcome, this approach cannot fully replace QALY-adjusted analyses. Despite these limitations, our findings provide valuable insights into the real-world effectiveness and cost implications of denosumab and ZA in managing bone metastases.

## 5. Conclusions

In conclusion, denosumab demonstrated superior efficacy in preventing SREs compared to ZA, particularly in breast and prostate cancer patients. However, its higher cost and the need for careful monitoring of calcium levels should be considered when making treatment decisions. Future prospective studies are needed to further evaluate the long-term benefits and cost-effectiveness of these treatments, especially in lung cancer patients. Overall, our findings contribute to the increasing body of evidence on the comparative effectiveness and cost-effectiveness of denosumab and ZA, providing valuable information for clinicians and policymakers.

## Figures and Tables

**Table 1:** Baseline demographic and clinicopathological findings.

Variables		All Patients *n =* 431	Denosumab *n =* 192	Zoledronic Acid *n =* 239	*p*-Value
Gender-Female *n* (%)		262 (60.8)	121 (63.0)	141 (59.0)	0.35
Tumour type *n* (%)					0.94
	Breast	233 (54.1)	105 (54.7)	128 (53.6)	
	Prostate	91 (21.1)	41 (21.4)	50 (20.9)	
	Lung	107 (24.8)	46 (24.0)	61 (25.5)	
Denovo bone metastasis *n* (%)		261 (60.6)	126 (65.6)	135 (56.5)	0.12
ECOG *n* (%)					0.41
	0	166 (38.5)	78 (40.6)	88 (36.8)	
	≥1	265 (61.5)	114 (59.4)	151 (63.2)	
Previously skeletal event *n* (%)		240 (55.7)	101 (52.6)	139 (58.2)	0.27
	Bone fractured	18 (4.2)	9 (4.7)	9 (3.8)	0.31
	Radiotherapy	228 (52.9)	97 (50.5)	131 (54.8)	
	Spinal cord compression	5 (1.2)	2 (1.0)	3 (1.3)	
	Bone surgery	5 (1.2)	1 (0.5)	4 (1.7)	
Cranial metastasis *n* (%)		103 (23.9)	38 (19.8)	65 (27.2)	0.08
Visceral metastasis *n* (%)		251 (58.2)	106 (55.2)	145 (60.7)	0.28
	Liver	179 (41.5)	71 (37.0)	108 (45.2)	0.41
	Lung	47 (10.9)	24 (12.5)	23 (9.6)	
	Other	23 (5.3)	11 (5.7)	12 (5.0)	
Median (range)					
Age of diagnosis (years)		57 (24–88)	57 (24–88)	57 (24–83)	0.33
Lead-time from diagnosis to MM (month)		54 (7–233)	73 (7–199)	49 (7–233)	0.44
Time to treatment initiation (day)		56 (0–3844)	65 (0–2506)	46 (0–3844)	0.02
Median duration of follow-up (month)		46 (6–328)	37 (6–250)	56 (6–328)	0.04

MM: Metachronous metastasis, *n:* number, ECOG: Eastern Cooperative Oncology Group.

**Table 2:** Univariate and multivariate logistic regression analysis for risk factors associated with skeletal-related events (SRE).

Variables		SRE (+)	SRE (−)	Univariate	*p*	Multivariate	*p*
		*n* (%)	*n* (%)	OR (95% CI)		OR (95% CI)	
Age (years)	<65	145 (46.9)	164 (53.1)	Ref.			
	≥65	46 (37.7)	76 (62.3)	0.6 (0.44–1.05)	0.08		
Tumor type	Prostate	51 (56.0)	40 (44.0)	Ref.		Ref.	
	Other	140 (41.2)	200 (58.8)	1.8(1.1–2.9)	0.01	1.9 (1.22–3.19)	0.005
ECOG	0	79 (47.6)	87 (52.4)	Ref.			
	≥1	112 (42.7)	150 (57.3)	0.8 (0.54–1.19)	0.27		
Prior SRE	No	97 (38.8)	153 (61.2)	Ref.		Ref.	
	Yes	94 (55.6)	75 (44.4)	1.6 (1.1–2.3)	0.01	1.6 (1.12–2.49)	0.011
Visceral Met	No	86 (37.9)	141 (62.1)	Ref.			
	Yes	105 (52.2)	96 (47.8)	1.2 (0.81–1.77)	0.34		
Cranial Met	No	170 (43.9)	217 (56.1)	Ref.			
	Yes	21 (46.7)	24 (53.3)	1.1 (0.72–1.76)	0.59		
Treatment	Denosumab	85 (44.3)	107 (55.7)	Ref.		Ref.	
	ZA	106 (44.4)	133 (55.6)	2.0 (1.36–2.97)	0.001	2.0 (1.34–2.98)	0.001

**Table 3:** Denosumab vs. Zoledronic Acid treatment intervention and cost analysis.

Cancer Type	Treatment	Median Intervention Months (Range)	*p*-Value	Mean ± SD Cost	Median Cost $ (Range)	*p*-Value
Overall	Denosumab	11 (2–74)	<0.001	1743.4 ± 1507.6	1155 (210–7770)	<0.001
	Zoledronic Acid	17 (2–237)		649.1 ± 830.9	374 (44–5214)	
Breast Cancer	Denosumab	17 (2–74)	0.002	2095.2 ± 1566.3	1680 (210–7770)	<0.001
	Zoledronic Acid	22 (2–237)		831.6 ± 946.3	484 (44–5214)	
Prostate Cancer	Denosumab	7 (3–39)	<0.001	1141.8 ± 1003.8	735 (315–4095)	0.002
	Zoledronic Acid	21 (3–191)		699.5 ± 770.4	451 (66–4356)	
Lung Cancer	Denosumab	9 (2–59)	0.084	1476.5 ± 1545.9	945 (210–6195)	<0.001
	Zoledronic Acid	5 (2–102)		224.9 ± 320.0	110 (44–2144)	

## Data Availability

The datasets analyzed during the current study are available from the corresponding authors.

## Supplementary Materials

The following supporting information can be downloaded at: https://www.mdpi.com/article/10.3390/jcm14186469/s1, Table S1: Subgroup analysis of skeletal-related event (SRE) incidence by cancer type and treatment; Table S2: Cost analysis of Denosumab and Zoledronic Acid by cancer type; Table S3: Primary and Sensitivity Analyses of SRE (Denosumab vs. Zoledronic Acid); Figure S1: Distribution of Denosumab and Zoledronic Acid costs (histograms); Figure S2: Boxplot comparing costs between Denosumab and Zoledronic Acid; Figure S3: Forest plots of univariate and multivariate logistic regression analysis for factors associated with skeletal-related events (SRE).
