# Supplementary material for: Comparative Efficacy and Cost-Effectiveness of Denosumab Versus Zoledronic Acid in Cancer Patients with Bone Metastases
Source: J Clin Med. 2025 Sep 14;14(18):6469. doi: 10.3390/jcm14186469 (PMC12470827; doi:10.3390/jcm14186469)
Supplement: Supplementary file 1 [file jcm-14-06469-s001.zip › jcm-3847872-supplementary.pdf]

Supplementary Material

Supplementary Table S1. Subgroup analysis of skeletal-related event (SRE) incidence by cancer type and treatment.

Cancer Type	Denosumab % (95% CI)	ZA % (95% CI)	p-value
Breast	31/105 (29.5%; 21.6–38.8)	63/128 (49.2%; 40.7–57.8)	0.002
Prostate	18/41 (43.9%; 29.9–59.0)	33/50 (66.0%; 52.2–77.6)	0.035
Lung	18/46 (39.1%; 26.4–53.5)	28/61 (45.9%; 34.0–58.3)	0.484

Values are presented as number of patients with SRE (percentage; 95% CI). CI = confidence interval.

Supplementary Table S2. Cost analysis of Denosumab and Zoledronic Acid by cancer type.

Cancer Type	Treatment	Median Intervention Months (range)	p-value	Mean ± SD cost	Median Cost $ (range)	p-value
Overall	Denosumab	11 (2 - 74)	< 0.001	1743.4 ± 1507.6	1155 (210 - 7770)	< 0.001
	Zoledronic Acid	17 (2 - 237)		649.1 ± 830.9	374 (44 - 5214)	
Breast Cancer	Denosumab	17 (2 - 14)	0.002	2095.2 ± 1566.3	1680 (210 - 7770)	< 0.001
	Zoledronic Acid	22 (2 - 237)		831.6 ± 946.3	484 (44 - 5214)	
Prostate Cancer	Denosumab	7 (3 - 39)	< 0.001	1141.8 ± 1003.8	735 (315 - 4095)	0.002
	Zoledronic Acid	21 (3 - 191)		699.5 ± 770.4	451 (66 - 4356)	
Lung Cancer	Denosumab	9 (2 - 59)	0.084	1476.5 ± 1545.9	945 (210 - 6195)	< 0.001
	Zoledronic Acid	5 (2 - 102)		224.9 ± 320.0	110 (44 - 2144)	

Values are presented as mean ± standard deviation (SD) and median (range). Costs were calculated based on unit prices of $105 per Denosumab dose and $22 per Zoledronic acid dose.

Supplementary Figure S1. Distribution of Denosumab and Zoledronic Acid costs (histograms).

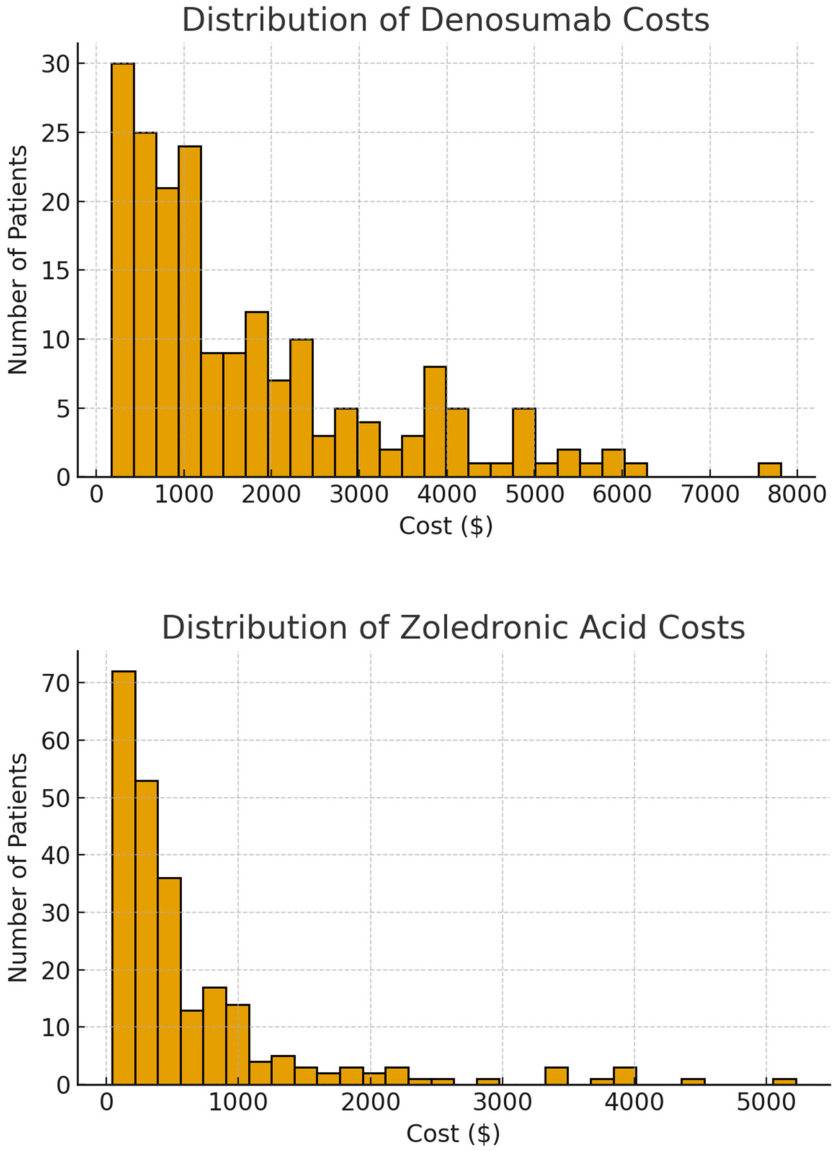

Supplementary Figure S2. Boxplot comparing costs between Denosumab and Zoledronic Acid.

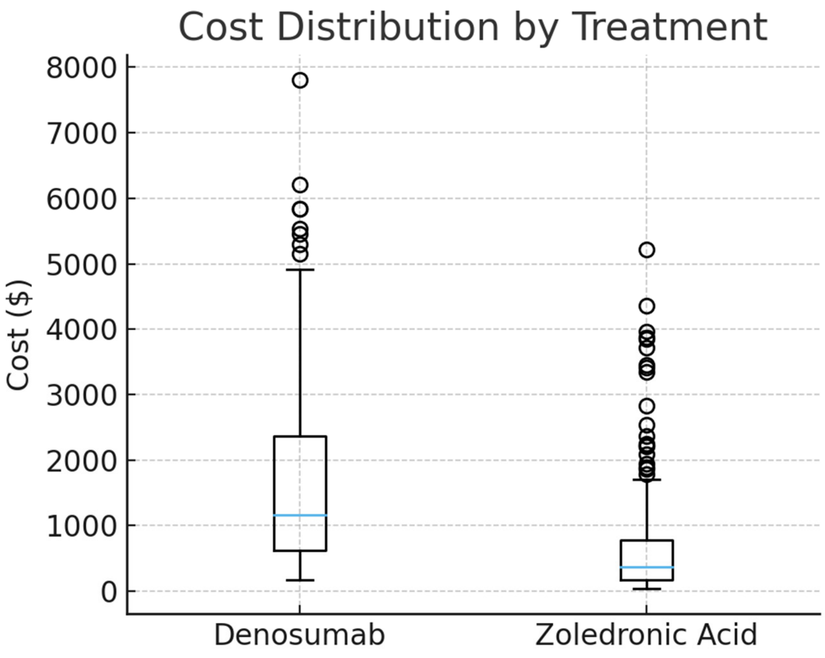

Supplementary Figure S3. Forest plots of univariate and multivariate logistic regression analysis for factors associated with skeletal-related events (SRE).

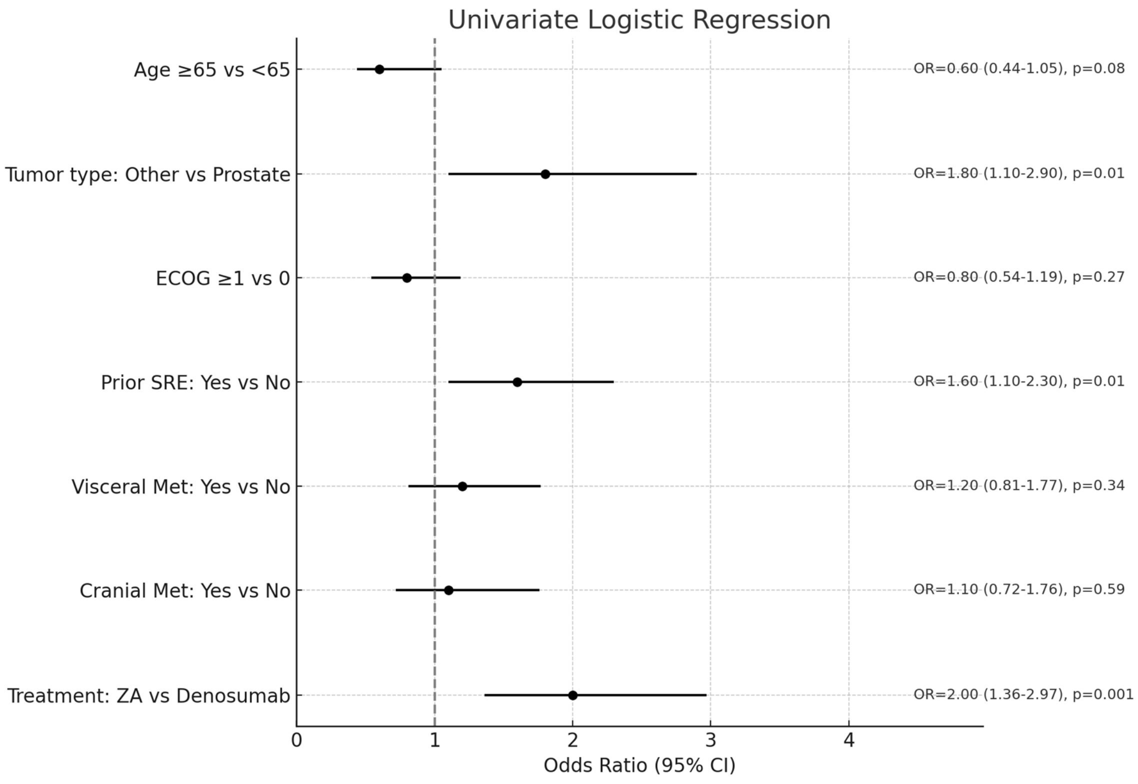

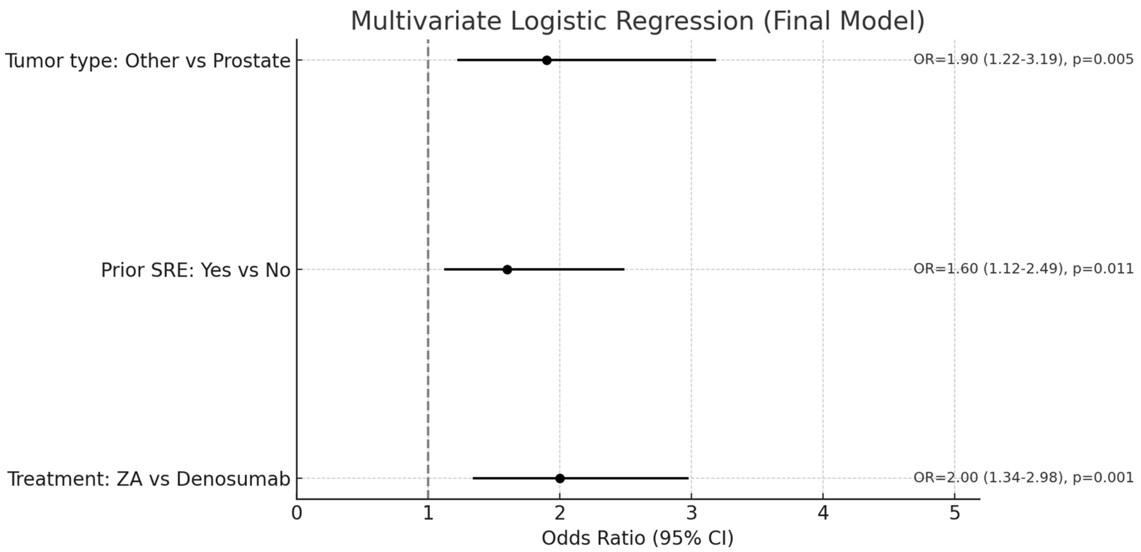

Supplementary Table S3. Primary and Sensitivity Analyses of SRE (Denosumab vs. Zoledronic Acid)

Analysis	Denosumab (N=192)	ZA (N=239)	Method	Result
Primary (full follow-up)	67 events (34.8%)	124 events (51.8%)	Chi-square test	p < 0.001
Sensitivity (≤37 m, Events)	34 events (21.6%)	42 events (20.1%)	Counts (KM estimate at 37m)	
KM cumulative SRE – 12 m	6.7%	4.8%	Kaplan–Meier	
KM cumulative SRE – 24 m	13.5%	12.4%	Kaplan–Meier	
KM cumulative SRE – 36 m	20.8%	19.5%	Kaplan–Meier	
KM cumulative SRE – 37 m	21.6%	20.1%	Kaplan–Meier (Log-rank)	χ^2=0.133, p=0.716
Cox regression (≤37 m)	Reference	HR 1.40	Cox proportional hazards	p = 0.149

Primary and sensitivity analyses comparing denosumab and ZA. The primary analysis used the entire follow-up (median follow-up: 37 months for denosumab and 56 months for ZA). To ensure comparable observation time, sensitivity analyses truncated follow-up at 37 months (the median follow-up of the shorter arm, denosumab), with events beyond this time censored.
